# FDG-PET findings of Ameloblastoma: a case report

**DOI:** 10.1186/s40064-015-0998-3

**Published:** 2015-06-11

**Authors:** Satoshi Seno, Kazuhiro Kitajima, Go Inokuchi, Ken-ichi Nibu, Tomoo Itoh, Yasuo Ejima, Ryohei Sasaki, Koji Sugimoto, Kazuro Sugimura

**Affiliations:** Department of Radiology, Kobe University Graduate School of Medicine, 7-5-2 Kusunoki-cho, Chuo-ku, Kobe, 650-0017 Japan; Department of Otolaryngology-Head and Neck Surgery, Kobe University Graduate School of Medicine, 7-5-2 Kusunoki-cho, Chuo-ku, Kobe, 650-0017 Japan; Department of Diagnostic Pathology, Kobe University Hospital, 7-5-2 Kusunoki-cho, Chuo-ku, Kobe, 650-0017 Japan; Department of Radiology, Division of Radiation Oncology, Kobe University Graduate School of Medicine, 7-5-2 Kusunoki-cho, Chuo-ku, Kobe, 650-0017 Japan

**Keywords:** ^18^F-fluorodeoxyglucose (FDG), PET, CT, MRI, Ameloblastoma

## Abstract

**Introduction:**

Ameloblastoma is a benign odontogenic neoplasm of the jaw, rarely presenting as a malignant tumor. Although it is very important to discriminate ameloblastoma from ameloblastic carcinoma in order to decide the appropriate operative procedure, this is difficult using conventional CT and MRI.

**Case descriptions:**

We report a case of maxillar ameloblastoma in a 78-year-old man where FDG-PET/CT was useful for making this discrimination. CT demonstrated a 31 × 43 × 46-mm mass in the left posterior maxillary sinus with destruction of its posterior and lateral wall and alveolar bone. MRI demonstrated a hypo- to isointense heterogeneous pattern on T1WI, heterogeneous hyperintensity with a prominent high-signal spot on T2WI, high signal intensity on DWI reflecting restricted diffusion, and strong heterogeneous enhancement. Because FDG-PET/CT showed mild FDG uptake (SUVmax 2.40) by the mass, ameloblastoma, rather than ameloblastic carcinoma, was considered to be the correct diagnosis.

**Discussion and evaluation:**

It appears that ameloblastic carcinoma shows intense FDG uptake, whereas ameloblastoma shows mild or moderate FDG uptake, and only rarely intense FDG uptake. Our experience suggests that FDG-PET/CT may be effective for discriminating ameloblastoma from ameloblastic carcinoma. Especially, in cases showing mild FDG uptake, benign ameloblastoma would seem the most likely diagnosis.

**Conclusions:**

FDG-PET/CT may be useful as an adjunctive modality for diagnosis, treatment planning and surveillance of ameloblastoma and ameloblastic carcinoma.

## Background

Ameloblastoma is a benign odontogenic neoplasm derived from ameloblastic epithelial tissue. It comprises only 1% of all jaw tumors and 11% of all odontogenic neoplasms (being the second most common) (Neville et al. 2002). The 2005 WHO histological classification of odontogenic tumors classified ameloblastoma into several types: solid/multicystic, extraosseous/peripheral, desmoplastic, and unicystic (Gardner et al. 2005). Although ameloblastoma is a benign neoplasm, it is locally aggressive and local recurrence is not rare (Inoue et al. 1988). Ameloblastic carcinoma is a term given to tumors that display histologically malignant features at both primary and metastatic sites with a pattern otherwise resembling ameloblastoma (Newman et al. 1995). To plan the most appropriate treatment, it is very important to determine whether a tumor is benign or malignant. However, discrimination between ameloblastoma and ameloblastic carcinoma is difficult by conventional morphological imaging modalities such as radiography, computed tomography (CT) and magnetic resonance imaging (MRI) (Devenney-Cakir et al. 2010, Dunfee et al. 2006, Matsuzaki et al. 2011, Minami et al. 1992).

Recently, assessment of glucose metabolism in cells using ^18^F-fluorodeoxyglucose (FDG) positron emission tomography (PET) has been used to identify aggressive tumors and to predict their degree of malignant potential. Here we describe a case of ameloblastoma with emphasis on the imaging features revealed by FDG-PET/CT.

## Case presentation

A 78-year-old male patient presented at the otolaryngology-head and neck surgery department of our hospital complaining of nasal bleeding. He had no pain, swelling or discharge. The patient gave consent to publish this case report and any accompanying images.

**Fig. 1:**
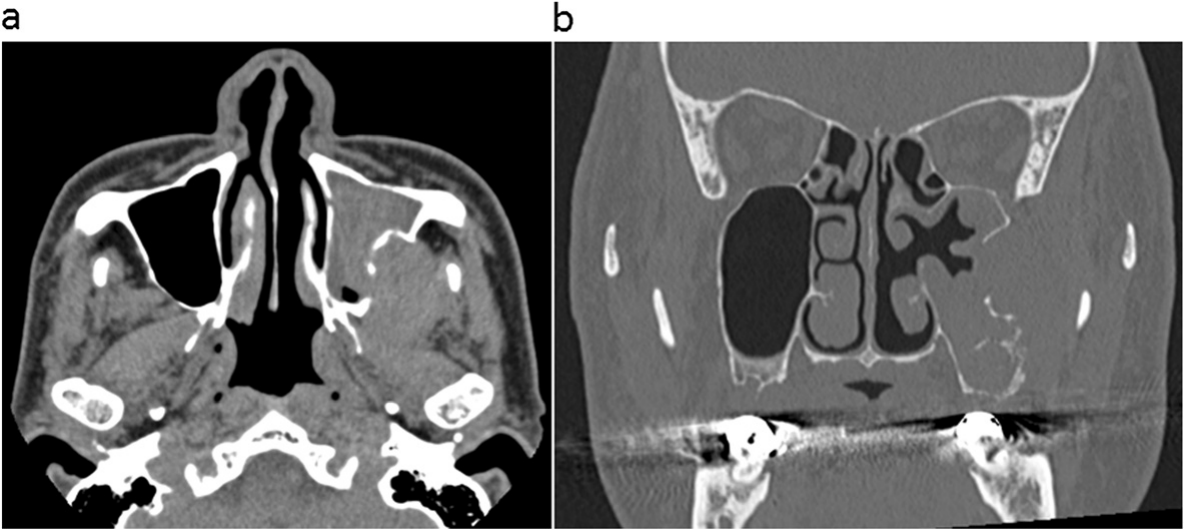
CT imaging findings. **a** Axial non-contrast CT image shows a 31 × 43 × 46-mm mass in the left maxillary sinus and masticatory space with resorption and destruction of the maxillary sinus posterior bone. **b** Coronal CT (bone-window) images show diffuse and partial resorption of the alveolar bone and elevation of the posterior maxillary sinus floor with destruction of the floor and lateral side wall

**Fig. 2:**
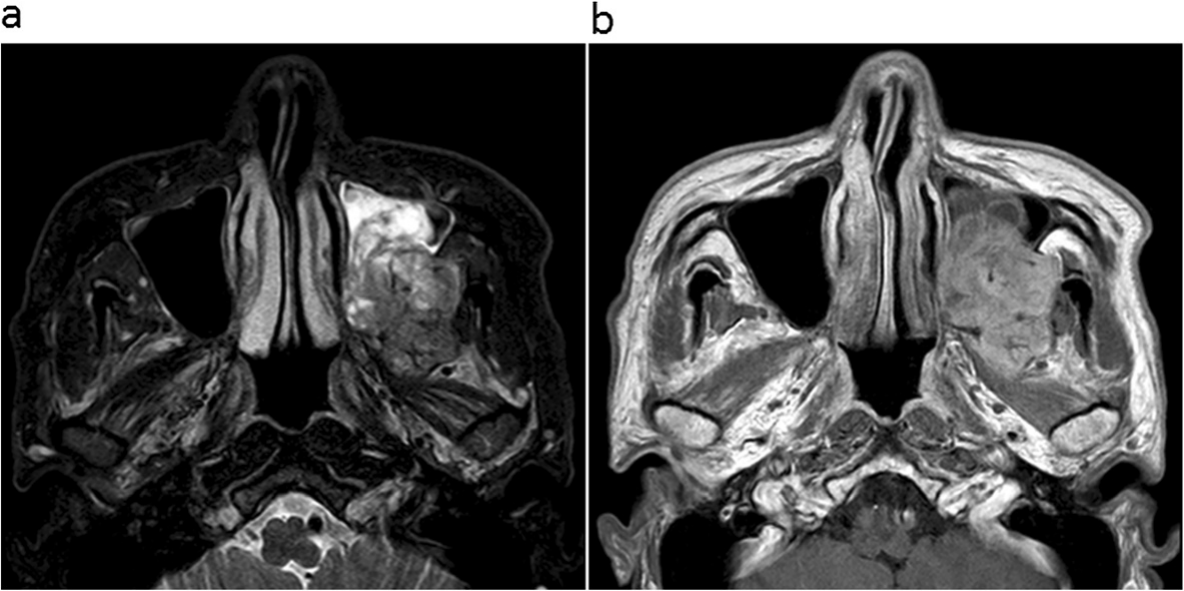
MRI findings. **a** Axial T2-weighted MR image shows heterogeneous hyperintensity with a prominent high-signal spot of the solid mass in the left maxillary sinus and masticatory space. **b** Axial contrast-enhanced T1-weighted MR image shows strong and heterogeneous enhancement of the mass

**Fig. 3:**
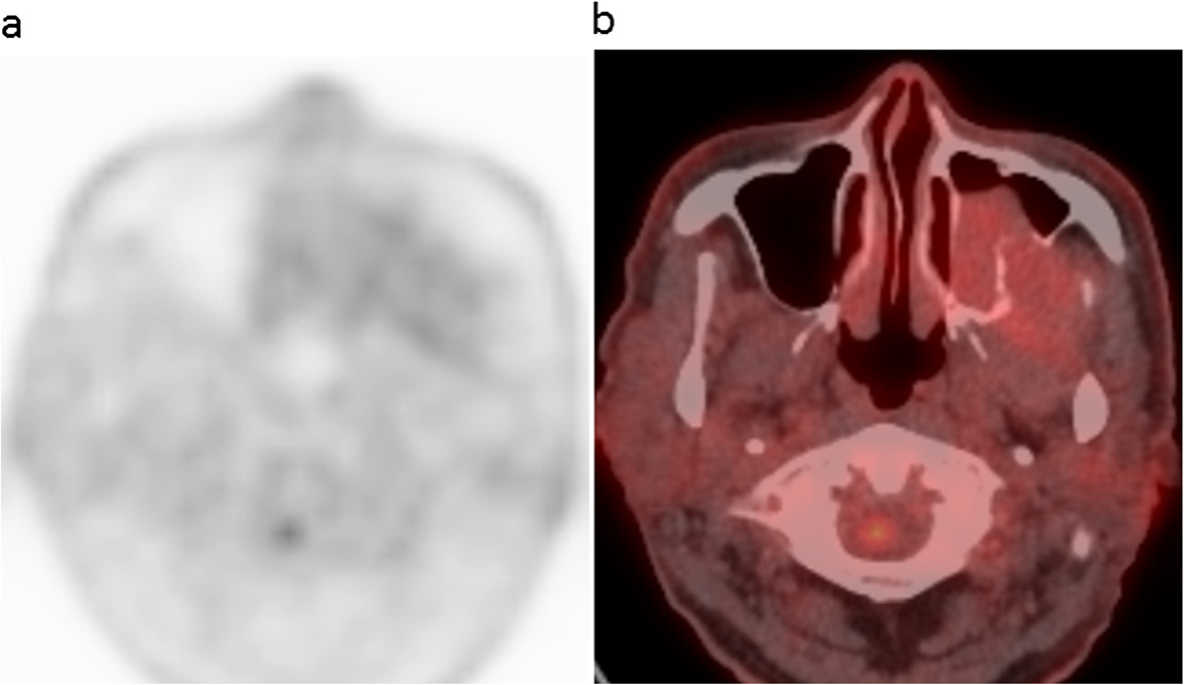
Findings of ^18^F-FDG PET/CT imaging. **a** PET and **b** fused PET/CT imaging show that the mass has mild FDG uptake with a maximum standardized uptake value (SUVmax) of 2.40

Non-contrast CT revealed a 31 × 43 × 46-mm mass in the left maxillary sinus and masticatory space, with posterior bone resorption and destruction of the maxillary sinus (Fig. 1a). The solid mass also showed partial resorption of the alveolar bone and elevation of the posterior maxillary sinus floor with destruction of the floor and lateral side wall (Fig. 1b). MRI was performed using a 1.5-T unit. MRI demonstrated hypo- to isointensity with a heterogeneous pattern on T1-weighted spin-echo images (repetition time/echo time (TR/TE) = 727/12 ms), heterogeneous hyperintensity with a prominent high-signal spot on T2-weighted fast spin-echo images (TR/TE = 4452/100 ms) (Fig. 2a), and high signal intensity on diffusion-weighted imaging (DWI) (b value = 0 and 1000 s/mm^2^), reflecting restricted diffusion. T1-weighted spin-echo images (TR/TE = 554/10 ms) enhanced with gadolinium-diethylene-triamine-pentaacetic acid (Gd-DTPA) (Magnevist: Bayer Schering Pharma, Osaka, Japan) showed strong and heterogeneous enhancement of the tumor (Fig. 2b). These findings of marginal bone destruction by the lesion on CT and MRI and abnormal signal intensity on DWI suggested a malignant tumor, and we first suspected ameloblastic carcinoma. The differential diagnosis included malignant peripheral nerve sheath tumor, rhabdomyosarcoma, pleomorphic adenocarcinoma or adenoid cystic carcinoma arising from the masticatory space. For initial staging, FDG-PET/CT was performed to investigate the presence of regional lymph nodes and distant metastasis. The mass showed mild FDG uptake with a maximum standardized uptake value (SUVmax) of 2.40 (Figs. 3a,b). On this basis, we considered the lesion to be a benign odontogenic tumor, probably ameloblastoma.

**Fig. 4:**
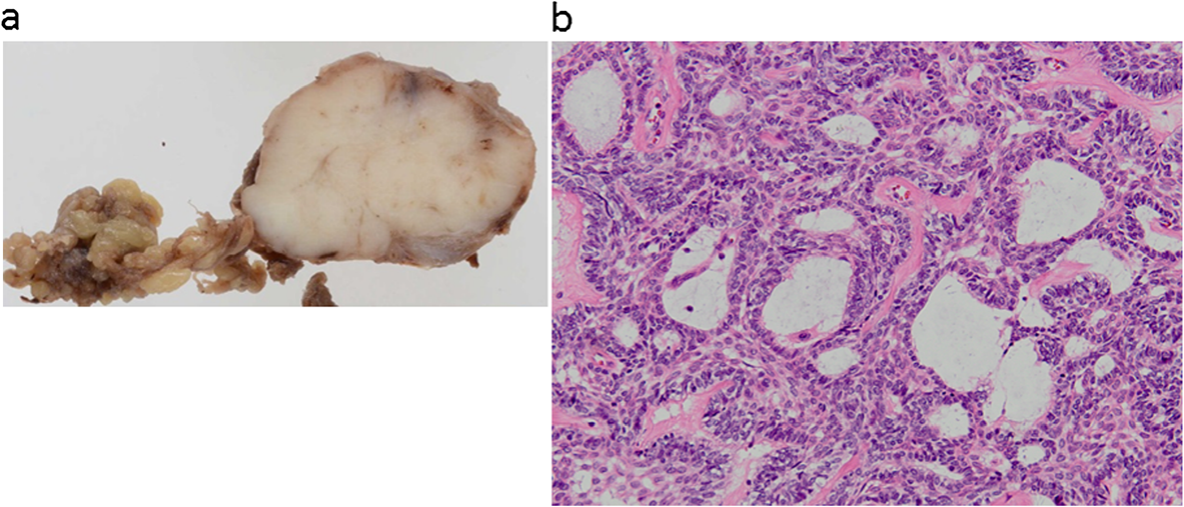
Histopathological findings. **a** Gross pathological view demonstrates a shiny white solid mass. **b** Microscopic view with hematoxylin and eosin staining reveals dense proliferation of follicular structures made up of tumor cells resembling odontogenic epithelium. Tall columnar cells resembling ameloblastoma cells surround peripheral follicles, which contain stellate reticulum-like central areas

We performed biopsy under general anesthesia and pathological examination of the sample confirmed ameloblastoma. Later the patient underwent partial mandibulectomy. Gross pathological review revealed a shiny white solid mass (Fig. 4a). Histopathological examination revealed dense proliferation of follicular structures made up of tumor cells resembling odontogenic epithelium. Tall columnar cells resembling ameloblastoma cells formed arrays surrounding peripheral follicles, which contained a stellate reticulum-like component in the central area (Fig. 4b). The pathological diagnosis was ameloblastoma.

At 2 years after surgery, there was no sign of tumor recurrence.

## Discussion

Ameloblastoma is a benign, locally aggressive and infiltrative odontogenic neoplasm with a high rate of recurrence. Ameloblastic carcinoma tends to be aggressive and has a potential for lymph node and distant metastasis. Therefore diagnostic imaging for initial staging before treatment is very important. Resection of mandibular masses is an extensive and potentially disfiguring type of surgery; therefore preoperative understanding of the extent and malignant potential of a tumor in this area is essential for achieving a curative and cosmetically acceptable result. However, the two neoplasms are morphologically similar (Devenney-Cakir et al. 2010, Dunfee et al. 2006, Matsuzaki et al. 2011, Minami et al. 1992), and therefore an additional imaging modality to achieve adequate discrimination is desirable.

Ameloblastoma and ameloblastic carcinoma most commonly occur in the posterior mandible, typically in the region of the third molar, with associated follicular cysts or impacted teeth. The slow growth of the tumor can lead to significant expansion of the mandible. Radiographically, both lesions can appear radiolucent, being either unilocular or multilocular, exhibiting a honeycomb-like appearance with tooth root resorption. Both lesions often have distinct borders, slight marginal sclerosis without periosteal new bone formation, loss of the lamina dura, resorption of the tooth apex and tooth displacement (Devenney-Cakir et al. 2010, Dunfee et al. 2006). CT findings include cystic areas of low attenuation with scattered isoattenuating regions, representating soft tissue components. The lesion can also erode through the cortex with extension into the surrounding oral mucosa (Dunfee et al. 2006). In addition, erosion of the roots of adjacent teeth is unique to ameloblastoma and indicates the aggressive behavior of the tumor. MRI demonstrates several common findings: multilocularity, mixed solid and cystic components, irregularly thick walls, papillary projections, and marked enhancement of the walls and septa (Dunfee et al. 2006, Minami et al. 1992). MRI is superior to conventional radiography and CT in demonstrating components of the tumor, extension within the bone marrow and involvement of adjacent extraosseous structures, which is important when considering surgical resection or reconstruction planning (Devenney-Cakir et al. 2010, Dunfee et al. 2006, Matsuzaki et al. 2011, Minami et al. 1992). Ameloblastic carcinoma generally exhibits more aggressive features such as dystrophic calcifications, cortical destruction, extraosseous extension and an extensive solid component.

Few reports have described the FDG-PET/CT features of ameloblastoma and ameloblastic carcinoma (Devenney-Cakir et al. 2010, Matsuzaki et al. 2011, Nguyen 2005, Otsuru et al 2008). Matsuzaki et al. reported one case of ameloblastic carcinoma of the maxilla demonstrating intense FDG uptake with a SUVmax of 28.3 (Matsuzaki et al. 2011). Otsuru et al. reported four cases of ameloblstoma (three arising from the maxilla and one from the mandible) showing mild to moderate FDG uptake, which was correlated with glucose transporter-1 (GLUT-1) expression (Otsuru et al 2008). Two groups have demonstrated that FDG-PET/CT was also useful for detection of metastases from ameloblastic carcinoma (Devenney-Cakir et al. 2010, Nguyen 2005). From these previous reports and the present case, it appears that ameloblastic carcinoma shows intense FDG uptake, whereas ameloblastoma shows mild or moderate FDG uptake, and rarely intense FDG uptake, however further analysis in a larger patient population is necessary to confirm this speculation. Low uptake in this case may be due to weak aggressiveness and invasiveness of this tumor. Our experience suggests that FDG-PET/CT may be effective for discriminating ameloblastoma from ameloblastic carcinoma. Especially, in cases showing mild FDG uptake, benign ameloblastoma would seem the most likely diagnosis. Moreover, as FDG-PET is useful for predicting the degree of malignant potential and for identifying ameloblastoma as a locally invasive tumor, prediction and early diagnosis of recurrent ameloblastoma, and personalized treatment in individual cases, may be possible (Otsuru et al 2008).

## Conclusions

We have reported the CT, MRI and FDG-PET/CT findings in a case of maxillar amelobastoma in a 78-year-old male patient. In this case, it was difficult to distinguish between amelobastoma and ameloblastic carcinoma by conventional CT and MRI. Mild FDG uptake on PET/CT was useful for diagnosis of amelobastoma. FDG-PET/CT may be useful as an adjunctive modality for diagnosis, treatment planning and surveillance of ameloblastoma and ameloblastic carcinoma.
